# Vertebral body and spinal cord infarction in a pile-driver operator with fibrocartilaginous disc embolism

**DOI:** 10.1016/j.clinme.2024.100226

**Published:** 2024-07-05

**Authors:** SJX Murphy, DJH McCabe, RL O Donohoe, AJ McCarthy

**Affiliations:** a Department of Neurology, Tallaght University Hospital/The Adelaide and Meath Hospital, Dublin, incorporating the National Children's Hospital (AMNCH), Dublin, Ireland; b Stroke Service, Tallaght University Hospital/The Adelaide and Meath Hospital, Dublin, incorporating the National Children's Hospital (AMNCH), Dublin, Ireland; c Department of Neurology, Chelsea and Westminster NHS Trust, London, UK; d Vascular Neurology Research Foundation, Tallaght University Hospital/The Adelaide and Meath Hospital, Dublin, incorporating the National Children's Hospital (AMNCH), Dublin, Ireland; e Department of Radiology, Tallaght University Hospital/The Adelaide and Meath Hospital, Dublin, incorporating the National Children's Hospital (AMNCH), Dublin, Ireland; f Department of Clinical and Movement Neurosciences, Royal Free Campus, UCL Queen Square Institute of Neurology, London, UK; g Department of Neurology, St Vincent's University Hospital, Dublin, Ireland; h Academic Unit of Neurology, School of Medicine, Trinity College Dublin, Ireland

## Abstract

We describe the case of a male heavy machinery operator who presented from work with a rapidly evolving spinal cord syndrome. Spinal MRI revealed thoracic vertebral body and cord infarction and evolving mild disc prolapse attributed to fibrocartilaginous disc embolism (FCDE). FCDE should be considered as one of the aetiological mechanisms of acute spinal cord infarction in pile-driver/heavy machinery operators, especially in association with adjacent vertebral body infarction and intervertebral disc prolapse. Magnetic resonance imaging (MRI) changes may evolve, warranting early follow-up MRI in appropriate cases.

## Report

**Fig. 1:**
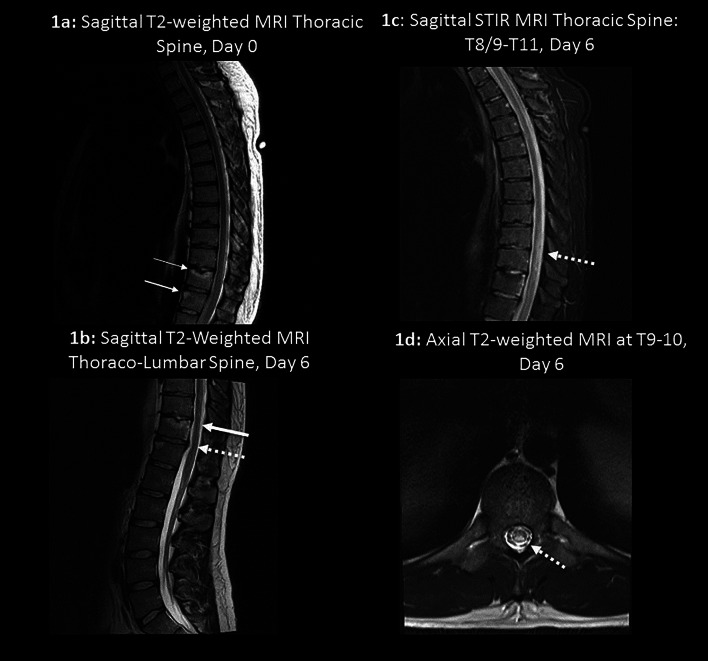
**a:** Schmorl's node and hyperintense endplate changes at T11 (thin arrow); hyper-intense endplate changes also at T12 (thicker arrow); **b:** Evolving hyper-intense posterior T11 vertebral body infarction (thick arrow); evolving mild T11–12 disc prolapse (dashed arrow). **c & d:** Evolving hyper-intense ‘posterior’ spinal cord infarction from T8/9T11 (dashed arrows).

A 28-year-old male pile-driver operator presented from work with acute, rapidly evolving bilateral leg weakness and urinary retention. Examination revealed normal cranial nerves, a mild spastic paraparesis, a T10 spinal sensory level to soft touch, pin-prick and temperature, but intact proprioception and vibration sensation. Serial spinal magnetic resonance imaging (MRI) over 6 days revealed evolving low thoracic spinal cord and vertebral body infarction, with increasing mild thoracic intervertebral disc prolapse, attributed to fibrocartilaginous disc embolism (FCDE), with early discordance between clinical findings of anterior spinal artery territory infarction and mainly posterior spinal cord signal changes on MRI (Fig. 1a–d). Comprehensive haematological, biochemical, microbiological, auto-antibody, and arterial and venous thrombophilia screening were normal. Cerebrospinal fluid examination, axial T1 fat-saturated MRI of neck arteries, CTA of extracranial arteries, thoracic and abdominal aorta, a trans-oesophageal echocardiogram and 24-h Holter monitor were normal. He was empirically commenced on aspirin monotherapy and medications for neuropathic pain.

Follow-up assessment 20 months after presentation revealed ongoing lumbar back pain, mild proximal leg weakness, allodynia below T9, with normal urinary sphincter function, constipation and erectile dysfunction. Cranial nerve examination remained normal. Limb examination revealed a mild spastic paraparesis (MRC grade 4/5 power in hip flexion bilaterally), normal coordination, lower limb hyperreflexia, with bilateral ankle clonus. Plantar responses were flexor on the right and extensor on the left, with lower limb hyperaesthesia below T8–T9 to soft touch, pin-prick and temperature, but normal proprioception and vibration sensation. He walked with a broad-based antalgic gait, but was functionally independent.

FCDE is a rare entity, may only be diagnosed post-mortem in some cases,^1^^,^^2^ and is proposed to arise from embolism of contents of the fibrocartilaginous nucleus pulposus of an intervertebral disc into the spinal vasculature.^3^ Schmorl's nodes are focal masses of fibrocartilage found within bony vertebrae, thought to result from degenerative disc disease^3^; their proximity to the vascular supply of the vertebral body makes them a risk factor for FCDE. In this case, the acute history, the patient's occupation and the neuroimaging findings of both evolving spinal cord and vertebral body infarction, in association with increasing T11–T12 disc prolapse, enabled establishment of this diagnosis and commencement of urgent treatment. We postulate that exposure to vibration to the spinal column provoked intervertebral disc prolapse and FCDE in the setting of a pre-existing Schmorl's node. FCDE-mediated spinal cord infarction may arise without vertebral body infarction on MRI; however, if both are present, this further supports FCDE as the aetiology of the stroke.^4^ Furthermore, this case illustrates that there may be early clinico-radiological discordance with respect to the predominant location of infarcts in the anterior vs. posterior thoracic spinal cord, but such discordance should not detract from the clinical diagnosis. To our knowledge, FCDE has not previously been reported in pile-driver operators, but must be considered in heavy machinery operators who present with an acute spinal cord syndrome with similar evolving neuroimaging findings. Importantly, if the first MRI is normal, repeat spinal cord MRI must be performed over the following days-week to look for dynamic changes.

## Funding

There was no targeted funding for this study. Prof McCabe's Vascular Neurology Research programme is currently supported by grants from The Meath Foundation, Ireland; The Adelaide Health Foundation, Ireland; The Vascular Neurology Research Foundation, Ireland; the Enterprise Ireland-Innovation Partnership Programme (co-funded by the 10.13039/501100008530European Regional Development Fund); and by unrestricted educational grant funding from Werfen, Spain; Sysmex Ireland-UK / Siemens, Germany; SINNOWA Medical Science & Technology Co., China; and Acquis BI Technology Ltd., Ireland. The authors report no disclosures relevant to this manuscript. None of the above charities or funding bodies had any influence on the design or content of this manuscript, or had any influence on the decision to submit the final manuscript for publication.

## Patient Consent

Written informed consent for publication was obtained from the patient.

## CRediT authorship contribution statement

**SJX Murphy:** Conceptualization, Investigation, Writing – original draft, Writing – review & editing. **DJH McCabe:** Supervision, Writing – review & editing. **RL O Donohoe:** Supervision, Writing – review & editing. **AJ McCarthy:** Supervision, Writing – review & editing, Writing – original draft.

## Declaration of competing interest

The authors declare the following financial support: Dominick McCabe reports that grant support for his Vascular Neurology Research Programme was provided by The Meath Foundation, Ireland; The Adelaide Health Foundation, Ireland; The Vascular Neurology Research Foundation, Ireland; and the Enterprise Ireland-Innovation Partnership Programme. Dominick McCabe reports that unrestricted educational grant funding for his Vascular Neurology Research Programme was provided by Werfen, Barcelona, Spain; Sysmex Ireland-UK and Siemens, Germany; SINNOWA, China; and by Acquis BI Technology, Ireland. None of the above charities or funding bodies had any influence on the design or content of this manuscript, or had any influence on the decision to submit the final manuscript for publication. The other authors declare that they have no known competing financial interests or personal relationships that could have appeared to influence the work reported in this paper.
